# The 2D:4D index is associated with the development of excess body weight in adults, but not with the rate of weight loss following bariatric surgery

**DOI:** 10.1038/s41598-022-12306-1

**Published:** 2022-05-16

**Authors:** Aleksandra Iljin, Bogusław Antoszewski, Tomasz Szewczyk, Aneta Sitek

**Affiliations:** 1 Department of Plastic, Reconstructive and Aesthetic Surgery, Medical University of Lodz, ul. Kopcińskiego 22, 90-153 Łódź, Poland; 2 Clinical Department of Gastroenterology, Oncology and General Surgery, USK No. 1, ul. Kopcińskiego 22, 90-153 Łódź, Poland; 3 Department of General Surgery, Regional Health Center, ul. Gen. Józefa Bema 5-6, 59-300 Lubin, Poland; 4 Department of Anthropology, Faculty of Biology and Environmental Protection, University of Lodz, ul. Banacha 12/16, 90-237 Łódź, Poland

**Keywords:** Medical research, Risk factors

## Abstract

2D:4D finger length ratio is a proxy of prenatal sex hormone exposure. Prenatal testosterone decreases and prenatal estrogens increase this index. In the current study we investigated whether the 2D:4D index, as a marker of the prenatal hormonal environment, is associated with the development of overweight and obesity in adults, and whether is it correlated with the rate of weight loss in patients after bariatric surgery. We tested 125 adults with obesity (BMI ≥ 30.0 kg/m^2^), 125 adults with overweight (BMI 25.0–29.9 kg/m^2^) and 153 persons with normal body weight (BMI < 25 kg/m^2^) of both sexes. We have found that the development of excessive body weight in men and women, and fat accumulation in the upper arms, thighs and lower legs in women with obesity (but not men) are associated with increased prenatal estrogen exposure. This relationship indicates a new area of activity in the field of obesity prevention. Moreover, it seems that the 2D:4D index (especially of the right hand) may be a useful factor in early prediction of the risk of developing excessive body weight in humans. The rate of weight loss after bariatric surgery is independent of prenatal exposure to sex hormones.

## Introduction

The 2D:4D ratio is the ratio of the length of the second (2D) to fourth (4D) finger of the hand. This indicator is determined in the prenatal period (between the 10th and 13th week of fetal life) under the influence of sex hormones^1^. The influence of prenatal testosterone (PT) and prenatal estrogens (PE) on digit ratio supported in experimental studies conducted in mice^2^. Moreover, a negative correlation between the PT/PE ratio in amniotic waters in pregnant women and 2D:4D ratio in children has been demonstrated^3,4^. Studies conducted in men also showed that genetically determined sensitivity to testosterone (polymorphism of the androgen receptor gene) is related to the digit ratio^5^. It is known that PT stimulates, while PE inhibits the growth of the fourth finger, therefore PT lowers and PE increases the size of 2D:4D and, as a result, it is on average lower in men than in women^2^. It has been estimated that the sexual dimorphism index for 2D:4D in adults is less than 5%^6^.

So far, it has not been clearly established whether 2D:4D changes with age in humans. Some authors did not demonstrate such changes^7,8^. In other studies a significant increase in the mean 2D:4D values of the left hand was found in girls and boys between the ages of one and nine, while in the older age groups the changes turned out to be much smaller^6^. On the other hand, on the basis of studies carried out in children aged 7–13 and repeated again after 4 years, slight but statistically significant changes of this indicator occurring in both sexes with age were less pronounced for the right hand, which is considered to be more sensitive to the influence of prenatal steroids^9^.

Obesity is a health problem with an epidemic status and the incidence of 15% for women and 11% for men; 13% in the general population^10^. Additionally, the frequency of this disease is gradually increasing^10^. Overweight and obesity arise as a result of excessive development of the fat mass in proportion to the fat-free body mass. It is believed that gender-specific physiological mechanisms are responsible for the higher incidence of obesity in women than in men. Women have more total fat which is deposited subcutaneously and in the gluteal/femoral regions, while men on average have less total body fat but more in the central/abdominal region^11^. According to researchers, the factor that allows women to store fat more efficiently is estrogen. The ability to effectively accumulate fat is an evident biological advantage for women due to the periods of pregnancy and lactation, which require high energy expenditure of the body^12^. Research shows that estrogens promote the accumulation of subcutaneous fat not only in women, but also in men^13–15^. They cause weight gain, mainly by inhibiting the hormonal activity of the thyroid gland and modulating the hypothalamus^16^. Moreover, it has been shown that binge eating is especially prevalent in the female sex and develops most often during adolescence, in combination with higher levels of estrogens^17^. In menopausal women, a decrease in estrogen levels increases total adipose tissue mass, reduces lean body mass^18^ and results in the loss of subcutaneous fat with a simultaneous increase in its amount in the abdominal cavity^19^. The mentioned effects of estrogen deficiency in the female sex can be eliminated by exogenous hormone replacement therapy^18,20^. In men, low testosterone levels lead to the accumulation of total and visceral fat mass, triggering a self-reinforcing cycle of complications that reduce the levels of this hormone. On the other hand, treatment with testosterone reduces fat mass, which has been verified in many randomized controlled clinical trials (RCT)^21^.

It therefore seems that a correct sex hormone profile is necessary to maintain a gender-specific (physiological) level of body fat. In addition, behavioral factors (physical activity, level of consumption, type of diet) are also important, affecting the maintenance of the body's energy homeostasis. It is known that all these elements are structurally controlled by prenatal sex hormones. Research indicates that in female mammals, including humans, deviations from normal exposure to sex hormones during fetal development cause ovarian dysfunction in adulthood^22^. Thus, disturbances in prenatal exposure to sex hormones, leading to disturbances in the postnatal hormone profile, can affect a number of feactures including, of course, BMI. Moreover, it is known that prenatal androgenization positively correlates with physical fitness in both sexes^23^ and with physical performance in females^24^, which clearly reduce BMI. High prenatal androgen levels in women have been associated with anorexia, while high prenatal estrogen levels have been associated with bulimia nervosa^25^. It has also been suggested that increasing global obesity incidence in both sexes is due to environmental estrogen-like substances^16^. Based on this, it can be assumed that v increased level of prenatal estrogen (resulting in higher 2D:4D values) will coexist with excess body weight (induced by estrogens through metabolic, endocrine and/or behavioral routes).

The effectiveness of weight reduction in the treatment of obesity depends on many variables, including the baseline BMI value, negative caloric balance, age, health status or family income^26^. In the case of bariatric treatment, the type of surgery performed may also play a role. Research indicates that gastric bypass (RYGB) causes greater weight loss than sleeve gastrectomy (SG), and RYGB and SG give better results than adjustable gastric bands (GB)^27^. Several studies have shown that the level of weight loss in obese people is sexually differentiated—men tend to lose more weight than women^12,28,29^. Moreover, it turns out that with a similar energy deficit, women lose less body fat than men^12^. It is believed that these differences may be influenced by a higher ratio of muscle mass to fat mass and higher resting and total energy expenditure in men than in women^12,29^, ie differences caused by sex hormones^12,18,21^. Based on this, it can be assumed that higher prenatal estrogenization (higher 2D:4D) will be associated with a lower rate of weight loss, especially fat tissue reduction.

Considering the multitude of mechanisms potentially linking prenatal sex hormone levels with human BMI in adulthood and weight loss after bariatric surgery (metabolic, hormonal, behavioral effects), this work was designed to investigate whether there is a relationship between 2D:4D (proxy index of prenatal exposure to PT/PE) and with the development of overweight and obesity in adults and whether it is associated with the rate of weight loss in patients after bariatric surgery. The authors expect to identify a positive relationship between 2D:4D and BMI, and a negative relationship with the rate of weight loss and its components after bariatric surgery, regardless of the sex of the subjects.

## Materials and methods

The study was approved by the Bioethics Committee of the Medical University of Lodz (RNN/105/18/EC) (Poland). We confirm that all research was performed in accordance with relevant guidelines/regulations. The study was conducted from July 1, 2017 to January 31, 2019. It comprised 125 obese adults (BMI ≥ 30.0 kg/m^2^), 125 overweight adults (BMI 25.0 to 29.9 kg/m^2^) and 153 persons with normal body weight (BMI < 25 kg/m^2^) of both sexes. The subjects were recruited from patients of the Clinic and Department of General Surgery (obese patients) and Plastic Surgery Clinic (patients with overweight and normal body weight) of the University Clinical Hospital No. 1 in Łódź (USK), (Poland).

Among obese patients there were 80 (23 men and 57 women) admitted to hospital for bariatric surgery (BMI 35.6–65.8 kg/m^2^), who underwent the following operations: laparoscopic sleeve gastrectomy (n = 45), laparoscopic adjustable gastric banding (n = 26), or laparoscopic Roux-en-Y gastric bypass (n = 9). Persons with normal weight or overweight came to the Plastic Surgery Clinic due to benign skin lesions or for scar treatment.

All patients gave informed and voluntary consent for participation in the study. Inclusion criteria comprised all adult patients of Polish origin, without defects and deformities in both hands who from July 1, 2017 to January 31, 2019 reported to the General Surgery Clinic or the Plastic Surgery Clinic or were admitted to the General Surgery Department of USK No. 1 in Łódź for surgical treatment of obesity, and who expressed written consent for participation in the study. The criterion of uniform origin of respondents was adopted in order to eliminate the inter-population variability in 2D: 4D. On the other hand, the criterion of the lack of deformation and defects in the area of the hands resulted from the specificity of the test, one of the main elements of which was the measurement of the length of the fingers.

### Anthropometric measurements

#### Study I

We recorded registered data (gender, age) and the height and weight, as well as the length of the index (2D) and ring (4D) fingers of both hands in all 403 patients (125 obese, 125 overweight and 153 persons with normal body weight). Measurements of body height (B-v) were made with an anthropometer with an accuracy of 1 mm, body weight was measured with a scale (TANITA BC-420 MA) with an accuracy of 0.1 kg, while the length of fingers was measured with a slider with an accuracy of 1 mm between the points *pseudophalangion* and *dactylion* (pph-da). Based on the length of fingers, the 2D:4D index for each hand and the difference between 2D:4D index in the right and left hand (Δ2D:4D = 2D:4DR – 2D:4DL) were calculated.

#### Study II

Two series of measurements were performed in 80 patients admitted to the hospital for bariatric surgery: on admission to hospital before surgery (time I) and about six months after surgery (time II). The mean length of break between tests performed at time I and time II was 186 days (min–max: 162–198 days). In each series, apart from the features listed in study I, the following components of body weight: FM (fat mass), FFM (fat-free mass) and MM (muscle mass), and TBW (total body water) were additionally measured using the body composition analyzer, which is an additional functionality of the scale (TANITA BC-420 MA).

The thickness of the skinfolds on both sides of the body was also measured: shoulder folds (vertical, on the posterior surface of the shoulders, in the middle of their length, with the arms hanging freely), subscapular folds (horizontal, below the inferior angle of the scapula), abdominal folds (oblique, measured on both sides at a quarter of the distance between the navel and the anterior superior iliac spine, counting from the navel), lower leg folds (vertical, at the center of the posterior aspect), and thigh folds (vertical, in the middle of the anterior and posterior aspects) are all measured using a skinfold caliper (Harpenden) with a 0.2 mm scale. In the case of bilateral measurements (except for 2D and 4D finger lengths), subsequent analysis involved averaged values for the right and left side.

### Intraclass correlation coefficient (ICC)

All measurements were carried out by one researcher between 08.00 and 12.00 h GMT to avoid diurnal variations in measurements. The measurement procedure was repeated by the same researcher after 15–20 min in a group of 60 people (30 with normal weight or overweight and 30 qualified for bariatric surgery). The reliability of the repeated measurements was assessed by calculating the intraclass correlation coefficients—ICC (2-way mixed-effects model, single measurement, absolute agreement)^30^.

### Statistical analysis

#### Study I

In the first stage of the analysis, using the height and weight, the BMI [kg/m^2^] of patients was calculated and then, on this basis, they were divided into three groups: with normal body weight (BMI < 25.0 kg/m^2^), overweight (BMI = 25.0–29.9 kg/m^2^) or with obesity (BMI ≥ 30.0 kg/m^2^). In each of the separated groups for women and men, the average values of age, body height, 2D and 4D finger length and 2D:4D of the right and left hand were calculated. The normality of distribution of each variable was tested using the Kolmogorov–Smirnov test. In the case of skewed distributions, the Box-Cox transformation was used.

Multiple regression was used for statistical analysis. The interactions between the independent variables were assessed using the General Linear Model (GLM). Corrected R^2^ and partial correlations were given for multiple regression. Eta square (η^2^) was given for interaction (GLM)^31^.

#### Study II

In the first step of data processing, differences in feature sizes between times I and II were calculated: Δ = x_I_ − x_II_ where: x_I_-feature size at time I, x_II_-feature size at time II. Next, the differences between consecutive measurements (Δ) were expressed as a percentage of the measurement of that feature pre-bariatric surgery: Δ% = Δ/x_I_*100%. Due to the fact that intervals between times I and II varied to some extent between patients, which could potentially affect the obtained results, percentage levels of reduction (Δ%) were expressed as daily percentage levels of reduction: DLR% = Δ%/Δt where: Δt-time (in days) between consecutive measurements at time I and time II. The correlations between 2D:4D and DLR% and between 2D:4D and anthropometric features of patients before bariatric surgery and 6 months after surgery were assessed by multiple regression.

All tests were two-tailed at a significance level of α < 0.05. All statistical analyzes were performed using the STATISTICA PL package. ver. 13.3.

## Results

### Intraclass correlation coefficient (ICC)

Based on the measurements performed twice in 60 patients, the intraclass correlation coefficient values were determined, which indicate excellent reliability: for B-v ICC = 0.997, for body weight (MC) ICC = 0.991, for 2DR ICC = 0.990, for 2DL ICC = 0.988, for 4DR ICC = 0.989, for 4DL ICC = 0.991. Slightly lower but still good reliability was obtained for 2D:4DR (ICC = 0.871) and 2D:4DL (ICC = 0.889). The lowest, although still acceptable, reliability was characterized by Δ2D:4D (ICC = 0.710). Due to the fact that the estimation of body composition and the measurements of the thickness of the skin and fat folds were performed only in patients qualified for bariatric surgery, the ICC for these measurements was calculated on the basis of repetitions made in a subgroup of 30 patients. Body composition measurements made using the BIA method stood out with excellent reliability: for FM ICC = 0.995, for FFM ICC = 0.999, for MM ICC = 0.998 and for TBW ICC = 0.996. The measurements of the brachial fold (ICC = 0.842), subscapular fold (ICC = 0.869), abdomen (ICC = 0.875), anterior thigh (ICC = 0.856) and posterior thigh (ICC = 0.850) had lower but still good reliability. The thickness of the skin-fat fold in the lower leg (ICC = 0.709) was of moderate reliability.

### Study I

#### Relationship between 2D:4D and Δ2D:4D indexes and sex and groups of BMI

The statistical characteristics of respondents are summarized in Table 1. Multiple regression analysis showed that 2D:4D of the right hand (R), adjusted for age and height, did not differentiate between men and women, but did vary depending on the BMI category. People with overweight and obesity had higher values of this index than people with normal body weight. All independent variables included in this analysis (age, height, gender and BMI groups) explained a total of 5.3% of 2D:4DR variability. The values of partial correlation suggest that obesity is slightly more related to 2D:4DR than overweight (partial correlation coefficient between obesity and 2D:4DR = 0.18, and between overweight and 2D: 4DR = 0.13). This means that the differences in the range of 2D:4DR between people with obesity and normal weight persons explain 3.24% of the variation in 2D:4DR unexplained by other factors (0.18^2^ = 0.0324), while differences between people with overweight and normal weight persons explain 1.69% of the variability of this index not explained by other factors (0.13^2^ = 0.0169) (Tab. 2). The lack of significant interaction between gender and BMI groups indicates that gender does not influence this 2D:4DR differentiation between individuals qualified for different BMI categories (Table 3).

**Table 1:** Mean age, anthropometric measures, 2D:4D ratios and Δ2D:4D of study participants.

Features	Males N = 239						Females N = 164					
	Normal body weight N = 96		Overweight N = 91		Obese N = 52		Normal body weight N = 57		Overweight N = 34		Obese N = 73	
	${\overline{\text{x}}}$	SD	${\overline{\text{x}}}$	SD	${\overline{\text{x}}}$	SD	${\overline{\text{x}}}$	SD	${\overline{\text{x}}}$	SD	${\overline{\text{x}}}$	SD
Age [yrs.]	44.34	12.52	49.36	18.04	49.11	13.23	47.50	10.71	48.18	11.83	42.46	11.73
B-v [cm]	181.0	6.8	178.4	7.8	176.7	6.3	164.8	7.0	162.8	6.9	165.0	5.7
2DR [mm]	74.1	3.9	73.6	4.2	73.5	4.6	69.6	4.5	70.7	4.0	69.7	3.5
4DR [mm]	76.6	4.0	75.2	4.7	74.8	4.9	71.1	4.8	71.9	4.1	70.2	4.0
2DL [mm]	74.4	3.4	73.3	4.1	73.8	4.3	70.0	4.3	70.9	3.7	69.8	3.5
4DL [mm]	76.3	4.1	75.1	4.6	74.7	4.8	70.6	4.8	71.6	4.1	69.8	3.8
2D:4DR	0.968	0.032	0.980	0.032	0.983	0.041	0.979	0.037	0.984	0.033	0.993	0.028
2D:4DL	0.975	0.028	0.976	0.030	0.989	0.033	0.993	0.032	0.992	0.036	1.001	0.026
Δ2D:4D	− 0.006	0.028	0.004	0.024	− 0.005	0.039	− 0.014	0.029	− 0.008	0.038	− 0.007	0.017

${\overline{\text{x}}}$, mean; SD, standard deviation; B-v, body height; 2D, second finger length; 4D, fourth finger length; 2D:4D, second to fourth digit ratio; R, right hand; L, left hand; Δ2D:4D = 2D:4DR -2D:4DL.

**Table 2:** Multiple regression analysis results concerning the relationship of 2D:4D ratios and Δ2D:4D with sex and BMI groups.

Independent variables		Dependent variables*								
		2D:4DR Corrected R^2^ = 0.0530			2D:4DL Corrected R^2^ = 0.0980			Δ2D:4D Corrected R^2^ = 0.0525		
		β	p	Partial correlation	β	p	Partial correlation	β	p	Partial correlation
Sex	Females vs Males	0.09	0.2498	0.06	**0.16**	**0.0344**	**0.11**	− 0.06	0.4432	− 0.04
BMI groups	Overweight vs normal body weight	**0.14**	**0.0119**	**0.13**	− 0.01	0.9211	− 0.00	**0.16**	**0.0034**	**0.15**
	Obesity vs normal body weight	**0.20**	**0.0003**	**0.18**	**0.15**	**0.0056**	**0.14**	0.07	0.1875	0.07

*Adjusted to age after Box-Cox transformation and body height.

2D:4D, second to fourth digit ratio; R, right hand; L, left hand; Δ2D:4D = 2D:4DR -2D:4DL; β, standardized regression coefficient; p, probability; Corrected R^2^, effect size; statistically significant relationships are marked in bold.

**Table 3:** Results of general linear model (GLM) of the significance assessor of interaction between sex and BMI groups for 2D:4D and Δ2D:4D variability.

Dependent variables*	Interaction: Sex × BMI groups		
	F	p	Effect size (η^2^)
2D:4DR	0.35	0.7041	0.0018
2D:4DL	0.15	0.8614	0.0008
Δ2D:4D	0.31	0.7316	0.0016

*Adjusted to age after Box-Cox transformation, body height, sex, and groups of BMI : normal, overweight, obesity.

F, test F; p, probability; η^2^, eta square.

The 2D:4D index of the left hand (L), after adjustment for age and height, was significantly higher in women than in men and higher in obese than in normal weight subjects. At the same time, this index did not differentiate between overweight and normal body weight persons. All independent variables (age, height, gender and BMI groups) together accounted for 9.8% of the variation in 2D:4DL. The association of obesity with 2D:4DL seems to be slightly stronger than the relationship between this index and gender. It should be noted that both these variables are very weak 2D:4DL correlates. Each of them alone explains only 1.2–2.0% of the 2D:4D variation unexplained by other factors (partial correlation: 0.11^2^ = 0.012; 0.14^2^ = 0.020) (Table 2). The interaction analysis showed that the relationship between 2D:4DL and BMI is not modified by gender (Table 3).

Δ2D:4D was significantly higher in overweight people than in people with normal body weight, but it did not differentiate obese people from the control group, as well as between women and men. All independent variables together explained 5.2% of the variability of Δ2D:4D, while overweight as an independent factor only 2.22% of the variability of this parameter (partial correlation 0.15^2^ = 0.022) (Table 2). The sex of the individuals did not modify the Δ2D:4D relationship with BMI (Table 3).

### Study II

#### Relationship between digit ratio (2D:4D) and the rate of weight and its components loss in patients after bariatric surgery

Table 4 summarizes the average values of body weight and its components as well as the thickness of the skin and fat folds in patients before bariatric procedures and six months after these procedures. The amount of reduction obtained after the operations was expressed as the difference between the measurements made at these two time points (Δ). The daily rate of reduction of individual traits is presented as DTR%. Due to the fact that the age of the patients and type of surgery affects ability to reduce body weight, the effect of these variables was controlled in the course of further analysis. It turned out that after adjusting the daily rate of weight and its components loss, as well as the thickness of the skin and fat folds on the patient's age and type of bariatric surgery these variables did not correlate statistically with 2D:4D of the right and left hand in the male and female patients (Table 5).

**Table 4:** Anthropometric characteristics of obese men and women undergoing bariatric procedures.

Features		Before bariatric treatment		6 months after bariatric treatment		Δ		DLR%	
		${\overline{\text{x}}}$	SD	${\overline{\text{x}}}$	SD	${\overline{\text{x}}}$	SD	${\overline{\text{x}}}$	SD
**Males n = 23**									
Body weight and its components [kg]	Body weight (BW)	146.89	15.13	113.02	20.50	33.87	15.65	0.11	0.05
	Fat mass (FM)	65.83	13.53	37.26	17.70	28.57	16.41	0.21	0.11
	Fat-free mass (FFM)	80.73	10.86	75.69	6.86	5.05	8.07	0.03	0.05
	Muscle mass (MM)	76.69	10.23	71.96	6.55	4.73	7.62	0.02	0.05
	Total body water (TBW)	60.91	7.97	54.66	4.87	6.25	7.22	0.04	0.06
Skinfolds [mm]	Shoulder	57.33	10.18	31.43	9.78	25.89	10.91	0.21	0.08
	Scapula	72.02	15.79	36.30	10.26	35.71	14.72	0.23	0.08
	Abdomen	84.64	9.01	44.78	12.40	39.86	13.77	0.22	0.08
	Calf	59.84	13.83	28.89	10.30	30.95	13.11	0.24	0.08
	Thigh (anterior aspect)	74.26	14.04	36.28	15.80	37.98	13.85	0.25	0.09
	Thigh (posterior aspect)	70.65	14.51	31.39	13.70	39.26	15.46	0.26	0.10
**Females n = 57**									
Body weight and its components [kg]	Body weight (BW)	122.01	17.57	93.20	15.91	28.81	11.48	0.11	0.04
	Fat mass (FM)	59.03	11.32	37.40	11.68	21.63	9.24	0.18	0.07
	Fat-free mass (FFM)	62.81	7.66	55.80	5.87	7.00	4.28	0.05	0.03
	Muscle mass (MM)	59.81	7.30	52.99	5.59	6.82	4.00	0.05	0.03
	Total body water (TBW)	46.54	6.07	40.25	4.64	6.28	3.39	0.06	0.03
Skinfolds [mm]	Shoulder	57.56	12.36	33.35	6.73	24.21	10.64	0.20	0.07
	Scapula	62.65	13.37	34.85	8.49	27.80	12.71	0.20	0.07
	Abdomen	76.15	14.21	41.11	9.61	35.04	14.08	0.22	0.08
	Calf	60.66	12.60	32.08	9.62	28.58	11.07	0.22	0.07
	Thigh (anterior aspect)	77.29	11.11	42.94	11.39	34.35	10.28	0.21	0.07
	Thigh (posterior aspect)	71.58	13.50	36.22	14.90	35.36	11.52	0.24	0.08

${\overline{\text{x}}}$, mean; SD, standard deviation; Δ, difference between measurements before bariatric treatment and 6 months after bariatric treatment; DLR%, daily percentage levels of reduction.

**Table 5:** Multiple regression analysis results concerning the relationship between the 2D:4D of the right and left hand and the rate of weight reduction and its components, as well as the thickness of the skin and fat folds.

Dependent variables DLR%*		Males n = 23				Females n = 57			
		2D:4DR		2D:4DL		2D:4DR		2D:4DL	
		β	p	β	p	β	p	β	p
Body weight and its components	Body weight (BW)	0.12	0.4282	0.14	0.4857	− 0.03	0.7856	0.04	0.7073
	Fat mass (FM)	0.01	0.9525	0.19	0.3515	− 0.03	0.7776	0.01	0.9522
	Fat-free mass (FFM)	0.27	0.2249	− 0.40	0.1798	− 0.14	0.3061	− 0.02	0.9090
	Muscle mass (MM)	0.28	0.2043	− 0.38	0.2030	− 0.03	0.8429	0.09	0.4890
	Total body water (TBW)	0.16	0.4723	− 0.42	0.1577	− 0.03	0.8424	0.09	0.4830
Skinolds	Shoulder	− 0.03	0.8938	− 0.22	0.4650	0.02	0.9241	− 0.02	0.9264
	Scapula	0.29	0.2604	0.27	0.2129	− 0.01	0.9335	− 0.07	0.5685
	Abdomen	− 0.04	0.8363	0.05	0.8317	0.09	0.4711	0.07	0.5561
	Calf	− 0.00	0.9958	− 0.19	0.5540	0.19	0.1571	0.22	0.1007
	Thigh (anterior aspect)	0.06	0.7895	0.00	0.9950	0.22	0.1196	0.20	0.1350
	Thigh (posterior aspect)	− 0.06	0.7968	− 0.12	0.6996	0.09	0.5156	0.06	0.6425

*DLR% adjusted for age after Box-Cox transformation and type of bariatric surgery.

β, standardized regression coefficient; p, probability.

The relationship between 2D:4D of the right and left hands and body composition was also investigated and fat folds in obese patients before and 6 months after bariatric surgery. Post-operative data were analyzed after adjustment for age, time from surgery to examination, and type of bariatric surgery. No correlation with 2D:4D R and L was found in men. In women 2D:4DR positively correlated with the thickness of the skin and fat folds on the arms, lower leg and anterior thigh measured before surgery. However, the strength of these correlations was weak (β, which in the case of simple linear regression is equivalent to the Pearson's r correlation coefficient, ranged from 0.28 to 0.36). After the bariatric procedure, none of the anthropometric parameters were related to 2D:4D of the right and left hand (Table 6).

**Table 6:** Multiple regression analysis results concerning the relationship between the 2D:4D of the right and left hand and anthropometric characteristics before and 6 months after bariatric surgery.

Features		Males n = 23				Females n = 57			
		2D:4DR		2D:4DL		2D:4DR		2D:4DL	
		β	p	β	p	β	p	β	p
**Before bariatric treatment**									
Body weight and its components	Body weight (BW)	− 0.01	0.9701	− 0.33	0.1223	0.06	0.6670	0.02	0.9081
	Fat mass (FM)	− 0.07	0.7517	− 0.09	0.6711	0.10	0.4500	0.05	0.7087
	Fat-free mass (FFM)	0.08	0.7189	− 0.34	0.1096	− 0.07	0.5908	− 0.09	0.4962
	Muscle mass (MM)	0.09	0.6721	− 0.32	0.1333	− 0.02	0.9015	− 0.04	0.7816
	Total body water (TBW)	0.19	0.3859	− 0.32	0.1378	− 0.00	0.9902	− 0.03	0.8272
Skinfolds	Shoulder	0.03	0.89301	− 0.104	0.50904	**0.36**	**0.0060**	0.20	0.1419
	Scapula	0.23	0.2811	0.23	0.3002	0.17	0.2014	0.08	0.5779
	Abdomen	0.25	0.2427	0.29	0.1861	0.18	0.1814	0.12	0.3796
	Calf	0.06	0.8025	− 0.05	0.8364	**0.28**	**0.0360**	0.18	0.1862
	Thigh (anterior aspect)	0.19	0.3794	0.04	0.8539	**0.32**	**0.0159**	0.13	0.3171
	Thigh (posterior aspect)	0.21	0.3367	0.15	0.4987	0.04	0.7479	− 0.00	0.9908
**6 months after bariatric treatment**									
Body weight and its components	Body weight (BW)*	− 0.06	0.8284	0.18	0.7064	0.06	0.627	− 0.00	0.9801
	Fat mass (FM)*	0.06	0.8331	− 0.05	0.911	0.08	0.5334	0.04	0.7542
	Fat-free mass (FFM)*	− 0.35	0.3863	0.71	0.2192	0.01	0.9179	0.14	0.5233
	Muscle mass (MM)*	− 0.35	0.3859	0.71	0.2183	0.01	0.9159	− 0.09	0.5258
	Total body water *TBW)*	− 0.13	0.6726	0.51	0.3408	0.02	0.8798	− 0.08	0.5607
Skinfolds	Shoulder*	0.22	0.5265	0.29	0.6289	0.04	0.7877	− 0.04	0.7619
	Scapula*	0.04	0.9078	0.03	0.9607	0.18	0.1896	0.2	0.1289
	Abdomen*	0.25	0.5203	− 0.15	0.7826	0.00	0.9923	0.02	0.8565
	Calf*	0.22	0.6057	− 0.16	0.8115	0.04	0.7825	0.04	0.7928
	Thigh (anterior aspect)*	0.35	0.5900	0.16	0.7629	0.03	0.8294	− 0.01	0.9441
	Thigh (posterior aspect)*	0.31	0.3635	0.01	0.9863	− 0.02	0.9008	0.03	0.7997

Significant values are in [bold].

*Adjusted for the age after Box-Cox transformation, time since surgery (in days) and type of bariatric surgery.

## Discussion

For the effective prevention/fight against obesity, it is important to recognize as fully as possible the factors and mechanisms that participate in its etiopathogenesis (including determination of prenatal conditions of this disease). In the present study, it was shown that higher values of the 2D:4D of the right and left hand (stronger prenatal estrogenization) are associated with the development of obesity and the higher values of 2D:4D of the right hand in addition to the development of overweight in both sexes. These results indicate that the development of excess body weight during postnatal ontogenesis is associated with exposure to elevated levels of estrogen in utero. This broadens the range of factors involved in the etiopathogenesis of obesity. Many other authors have also demonstrated that 2D:4D not only of the right hand, but also of the left hand, positively correlates with body mass index (BMI) in adults of both sexes^32,33^. Other research revealed a positive relationship between BMI and 2D:4D, but only for the right hand^34,35^. On the other hand, Fink et al. found that BMI in men (but not in women) is positively related to the digit ratio, but it remains significant only for the left hand^36^. Contrary to this report, Manning et al. indicate that 2D:4D is positively associated with BMI, but only in women^37^.

There are also studies that do not show the existence of such a relationship in young women^37,38^ as well as young women and men^39–42^. On the other hand, others reveal an inverse correlation between 2D:4D and BMI^43^. Similarly, Klimek et al., based on studies performed in men, indicate that low 2D:4D (stronger prenatal androgenization) is associated with higher body weight in childhood and adolescence^44^. At the same time, other studies of children and adolescents from various populations did not confirm the relationship between 2D: 4D and relative body weight^45,46^.

According to the literature data, other anthropometric features and indices associated with excess body weight may also correlate with digit ratios. An example of such a feature is the circumference of the neck (NC), which is considered a simple measure for the identification of obesity^39,47^. Research shows that it may be positively correlated with 2D:4D^32,47^, negatively correlated^43^ or not related^48^. In the case of waist circumference (WC) and hip circumference (HC) and their relationship—WHR researchers report both positive^32^ and negative associations^36,38,40,43^ with right and left hand 2D: 4D. Others, on the other hand, report a lack of association with 2D: 4D^41,48^. Another indicator of nutritional status is the waist-to-body height ratio (WHtR). Here, too, mixed results have been reported. Some authors point to positive associations between 2D:4D of the right and left hand and WHtR^32^, others suggest negative correlations between these variables^38,41^. Additionally, statistically significant differences were found in 2D:4D for both right and left hands depending on the category of waist circumference and WHtR. Researchers have reported that women with abdominal obesity have the lowest digit ratio values^38^. This is in line with reports suggesting that testosterone promotes fat accumulation in the waist area^11^. On the other hand, the study by Muller et al.^49^, based on a large sample of over 24 thousand women and 17 thousand men aged 40–69 years, did not reveal any significant relationship between 2D:4D of the right and left hand and measurements of adiposity (weight, BMI, waist circumference, hip circumference, waist to-hip ratio, fat-free mass or fat mass).

The above cited publications show a large discrepancy in the results concerning the relationship between 2D:4D and the development of excessive body weight in humans. Our results support the idea that prenatal estrogenization (high 2D:4D) promotes the development of obesity in both sexes. This is indicated by higher 2D:4D values of the right and left hand in people with obesity than in people with normal body weight. This result is consistent with the reports of many other authors^32–34,36,37^ and indicates a new area of activity in the prevention of this disease. At the same time, it seems that the 2D:4D index (especially of the right hand) may be a useful factor in early prediction of the risk of excessive body weight development in humans.

Our result suggesting a relationship between prenatal estrogenization and high BMI is consistent with the report by Gülge et al. indicating that higher 2D:4D values of both hands coexist with greater fat mass and total fat and smaller fat free mass in the total group of women and men^33^. Similar results were obtained by Pruszkowska-Przybylska et al., proving that in girls aged 6–13 years, higher 2D:4D values are accompanied by a lower percentage of muscle mass (MM%). However, this effect was not found in boys^50^. Moreover, Bakholdina et al. report that 2D:4D is positively correlated with the thickness of the forearm and abdomen skin folds, but only in young women^41^. Bakholdina's results agree with Fink's observations who stated an association between the 2D:4D ratio and body shape only in females^36^.

Our study did not confirm the relationship between 2D:4D and body weight and its components (FM, FFM, MM, TBW), but showed that in women with obesity, the right 2D:4D is positively correlated with the thickness of the skin and fat folds in some areas of the body (on the shoulders, thighs and lower legs). It is worth noting that the 2D:4D index of the right hand is considered a better indicator of prenatal exposure to sex hormones than the corresponding index of the left hand^9,51^. Moreover, the skin and fat folds reflect the amount of subcutaneous fat, which accumulates more easily in women than in men, and it is favored by estrogens^11^. Additionally, the indicated places (especially the thighs) belong to the areas where fat is more easily deposited in the female than in the male sex, and this tendency is also related to the level of estrogens^11^. Our results therefore suggest that prenatal estrogens also play a role in the formation of subcutaneous fat deposits at sites typical for the female sex. At this point it is worth emphasizing that the relationships of prenatal sex hormones with morphological features (nutritional status indices) can be realized through many mechanisms, including programming of metabolism^52^, postnatal hormonal functionality of gonads^22^ and behavioral factors such as for example the level of physical fitness^23^ and physical performance^24^.

Another goal of our work was to investigate whether 2D:4D is related to weight loss and its components, as well as fat folds in patients after bariatric surgeries. Due to the fact that adipose tissue is reduced faster in men than in women^12,28,29^, we expected that lower 2D:4D values (stronger prenatal androgenization) may be associated with a higher rate of weight loss and/or its components (FM, FFM, MM, TBW) and a higher rate of reduction of skinfolds after such treatments. Our results, however, did not confirm the relationship between 2D:4D of the right and left hand and the rate of reduction of these parameters in men and women. This result supports the thesis that this process is not conditioned by the level of prenatal sex hormones.

Finally, we would also like to refer to 2D:4D sexual dimorphism. In our study, we found it only in the case of 2D:4DL, which was characterized by higher values in women than in men. The direction of these differences is consistent with numerous reports on other populations^7,33,36,40–43^. Surprisingly, we did not identify statistically significant differences between men and women with regard to 2D:4DR, although the empirical differences for this indicator were as expected (it was, on average, lower in men than in women). In most publications, the authors indicate that the digit ratio of the right hand is a dimorphic feature^7,33,36,40–43^. Therefore, we expected to find such differences in relation to 2D:4DR also in our study. It seems that the lack of dimorphism in the 2D:4D range of the right hand may be related to the body weight. If the tendency to accumulate adipose tissue is associated with higher values of 2D:4D (prenatal estrogenization), this effect may be eliminated by 2D:4D sexual dimorphism, especially in the right hand, which is more strongly related to fetal sex steroids than the left hand^3,51^.

## Conclusions

High estrogen exposure during prenatal development is significantly related to the development of excessive body weight in men and women and the accumulation of subcutaneous fat in the arms, thighs and lower legs in women with obesity. This relationship indicates a new area of activity in the field of obesity prevention. Moreover, it seems that the 2D: 4D index (especially of the right hand) may be a useful factor in early prediction of the risk of developing excessive body weight in humans.

The rate of weight loss and its components reduction after bariatric surgery is not related to the level of prenatal exposure to sex hormones in both women and men.

### Limitation

This study is a cross-sectional study, which has limitations in inferring causality, and the related behavioral factors such as diet and physical activity related to obesity are not controlled. Also, we did not analyze the body composition and thickness of the skin and fat folds in people with a BMI < 30 kg/m^2^. The sample size is also a limitation.
